# Infliximab against severe COVID-19-induced cytokine storm syndrome with organ failure—a cautionary case series

**DOI:** 10.1186/s13054-020-03158-0

**Published:** 2020-07-17

**Authors:** Andreas Stallmach, Andreas Kortgen, Falk Gonnert, Sina M. Coldewey, Philipp Reuken, Michael Bauer

**Affiliations:** 1 Department of Internal Medicine IV (Gastroenterology, Hepatology, and Infectious Diseases), Jena University Hospital, Am Klinikum 1, 07747 Jena, Germany; 2 Department of Anesthesiology and Intensive Care Medicine, Jena University Hospital, Am Klinikum 1, 07747 Jena, Germany; 3 Department of Anesthesiology and Intensive Care Medicine, SRH Wald-Klinikum Gera, Straße des Friedens 122, 07548 Gera, Germany; 4 Septomics Research Center, Jena University Hospital, 07745 Jena, Germany; 5 Center for Sepsis Control and Care, Jena University Hospital, Am Klinikum 1, 07747 Jena, Germany

The severe acute respiratory coronavirus 2 (SARS-CoV-2) pandemic has prompted search for therapeutics tackling both the pathogen and the overwhelming host response, on an unprecedented scale. In a minority of patients, the disease may cause frequently lethal complications from acute respiratory distress syndrome to multisystem organ failure presumably driven by a cytokine storm [1]. Therefore, anti-cytokine therapies may be helpful to prevent tissue injury. However, these anti-inflammatory drugs constitute double-edged swords; while they can prevent organ damage, they increase the risk of concomitant (super)infection.

Recently, Neurath argued for a protective effect of tumor necrosis factor (TNF) inhibitors in severe COVID-19 [2]. Specifically, TNF may aggravate lymphopenia through direct killing via TNF/TNFR1 signaling in T cells [3], and T cell dysfunction reflects an important yet underestimated target for immunomodulatory interventions [4]. Thus, anti-TNF strategies may be an interesting option in severe COVID-19. This is supported by data from patients with inflammatory bowel disease (IBD) already on anti-TNF treatment. As of June 16, 2020, outcome data from 1511 IBD patients with COVID-19, among them, 433 patients on anti-TNF, are available from the SECURE-IBD registry [5]. Of these, 16% were hospitalized but only three patients (0.7%) died. Compared to other anti-inflammatory drugs such as steroids, thiopurines, or JAK inhibitors from SECURE-IBD, the safety signal of anti-TNF therapy seems simply the best.

These positive clinical data in the absence of a causative therapy encouraged us to use infliximab (IFX), a chimeric monoclonal anti-TNF antibody, as an experimental therapy in patients with severe COVID-19 in the absence of IBD. In this case series, we retrospectively explore the possible impact of IFX in critically ill patients with confirmed COVID-19 in comparison with a contemporaneous group of COVID-19 receiving supportive therapy only. Seven patients (47–70 years; one woman) were treated with a single infusion of IFX (5 mg/kg body weight) between 0 and 3 days after admission. One of the six had no known preexisting medical conditions apart from obesity, which was present in all cases (Table 1).

**Table 1:** Comparison between infliximab and non-infliximab

	Infliximab (*n* = 7)	Non-infliximab (*n* = 17)
Age (years)	60 (47–66)	66 (42–91)
Sex (m/w)	6/1	11/7
BMI (kg/m^2^)	32.4 (24.0–36.1)	28.7 (22–45)
ICU admission (*n*)	6 (86%)	15 (88%)
Death related to COVID-19	1 (14.2%)	6 (35.3%)
Mechanical ventilation (*n*)	3 (42.8%)	9 (52.9%)
ECMO (*n*)	2 (28.6%)	1 (5.9%)
RR syst (mmHg)	110 (100–125)	128 (94–174)
AF (1/min)	24 (18–39)	27 (16–42)
HF (1/min)	72 (61–120)	84 (60–127)
SpO_2_ (%)	91 (89–96)	92 (84–99)
Temperature (°C)	37.7 (36.2–37.7)	37.1 (36.7–39.4)
WBC (Gpt/l)	8.9 (3.4–16.4)	7.0 (2.2–13)
CRP (mg/l)	178.1 (113.4–401.3)	187 (18.7–323)
PCT (ng/ml)	0.28 (0.06–15.99)	0.58 (0.1–11)
Ferritin (μg/l)	2777.4 (697–70,693.8)	1453.0 (124.7–7391)
d-dimer (μg/l)	683 (10.2–48,588)	475.5 (146–8015)
LDH	10.6 (8.23–131.65)	6.54 (3.31–11.8)
Creatinine (mmol/l)	92.0 (59–303)	77.5 (55–384)^a^
ALAT (μmol/l*s)	2.79 (0.62–3.19)	0.55 (0.1–2.93)

Data are presented as absolute number and percentage or as median and range

^a^Excluding patients with chronic hemodialysis

In all IFX-treated patients, a rapid and at least temporary decrease of proinflammatory cytokines such as IL-6 (see Fig. 1) and other inflammatory markers (CRP, LDH) was observed along with clinical improvement in six of seven patients. Lymphocyte count (available in six of seven patients) before IFX infusion was initially below 1 Gpt/l and increased in five patients after IFX (Fig. 1b). The 17 patients of the control group showed 35% overall mortality at a similar stage of hospitalization along with prolonged systemic inflammation.

**Fig. 1:**
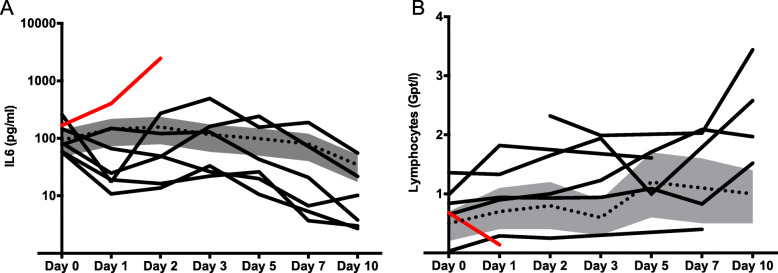
Impact of TNF neutralization by infliximab on the course of IL-6 (**a**) and lymphocytes (**b**) in severe COVID-19. Gray bar and dotted lines indicate Q1 and Q3 and median of 15 contemporary control patients, respectively. Solid lines indicate the individual course of seven patients treated with infliximab. Red: course of the non-survivor receiving infliximab

Proinflammatory cytokines driving the pathogenesis of IBD, such as TNF-α or IL-6, are similarly increased in the inflammatory response to SARS-CoV-2 and are associated with poor outcomes [3]. Therefore, early anti-inflammatory therapies carry the potential to avoid deterioration of organ function while the potential after the manifestation of an exuberant inflammatory response is probably limited. Concerning this, we observed one death out of seven patients subjected to IFX therapy. This patient demonstrated severe procoagulant activity with recurrent episodes of fulminant thromboembolic events in the pulmonary circulation and exorbitant increased ferritin levels (70,694 μg/l) as a marker of progressive hyperinflammation already prior to IFX.

The limited sample size and the uncontrolled study design preclude definitive statements. Nevertheless, this initial experience is promising, and anti-TNF strategies warrant to be assessed in randomized controlled trials. As such, data from an RCT with adalimumab (together with antiviral drugs), currently enrolling patients with severe COVID-19 in China, are eagerly awaited albeit “theranostic” stratification, and identification of the target population might require more attention for anti-inflammatory strategies [1].

## Data Availability

Not applicable.
